# Design of a multi-epitope vaccine against *Haemophilus parasuis* based on pan-genome and immunoinformatics approaches

**DOI:** 10.3389/fvets.2022.1053198

**Published:** 2022-12-29

**Authors:** Maonan Pang, Teng Tu, Yin Wang, Pengfei Zhang, Meishen Ren, Xueping Yao, Yan Luo, Zexiao Yang

**Affiliations:** ^1^College of Veterinary Medicine, Sichuan Agricultural University, Chengdu, China; ^2^Key Laboratory of Animal Diseases and Human Health of Sichuan Province, Chengdu, Sichuan, China

**Keywords:** *Haemophilus parasuis*, reverse vaccinology, pan-genome analysis, multi-epitope vaccine, immunoinformatics

## Abstract

**Background:**

Glässer's disease, caused by *Haemophilus parasuis* (HPS), is responsible for economic losses in the pig industry worldwide. However, the existing commercial vaccines offer poor protection and there are significant barriers to the development of effective vaccines.

**Methods:**

In the current study, we aimed to identify potential vaccine candidates and design a multi-epitope vaccine against HPS by performing pan-genomic analysis of 121 strains and using a reverse vaccinology approach.

**Results:**

The designed vaccine constructs consist of predicted epitopes of B and T cells derived from the outer membrane proteins of the HPS core genome. The vaccine was found to be highly immunogenic, non-toxic, and non-allergenic as well as have stable physicochemical properties. It has a high binding affinity to Toll-like receptor 2. In addition, *in silico* immune simulation results showed that the vaccine elicited an effective immune response. Moreover, the mouse polyclonal antibody obtained by immunizing the vaccine protein can be combined with different serotypes and non-typable *Haemophilus parasuis in vitro*.

**Conclusion:**

The overall results of the study suggest that the designed multi-epitope vaccine is a promising candidate for pan-prophylaxis against different strains of HPS.

## Introduction

**Figure 1:**
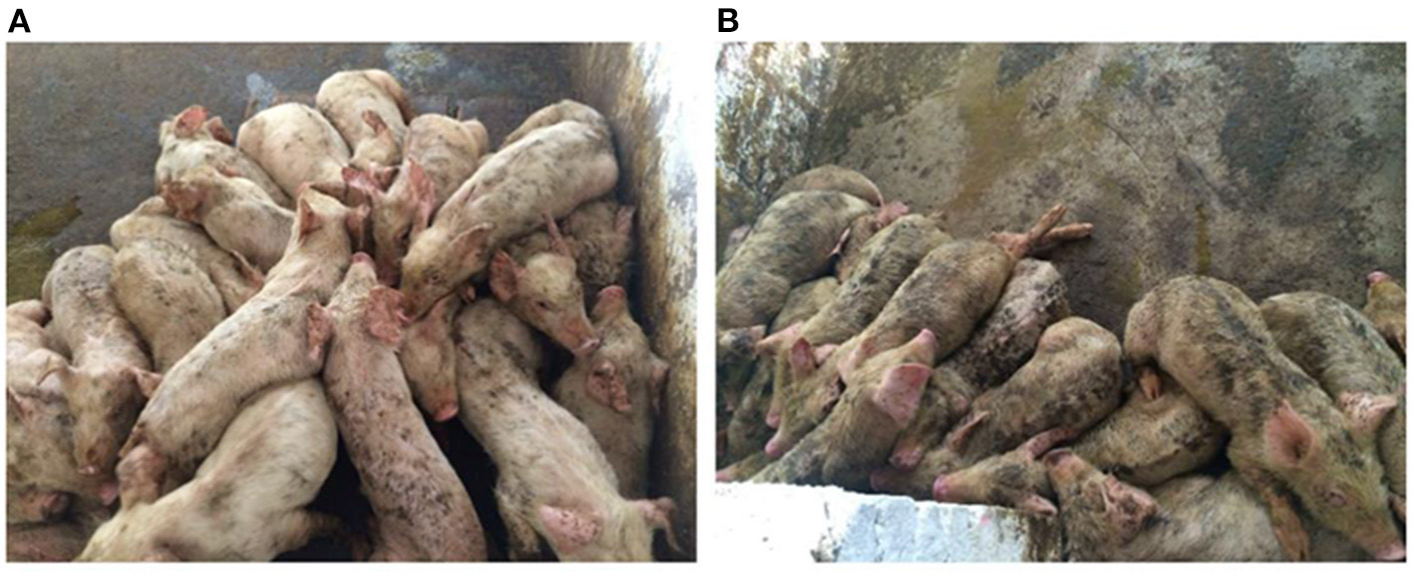
Group of nursery pigs diagnosed with Glässer's disease. **(A,B)** Pigs gather in the corner of the pen to protect themselves from the cold, their bodies are dirty, and their coats are ragged. Photographs taken by the author.

**Figure 2:**
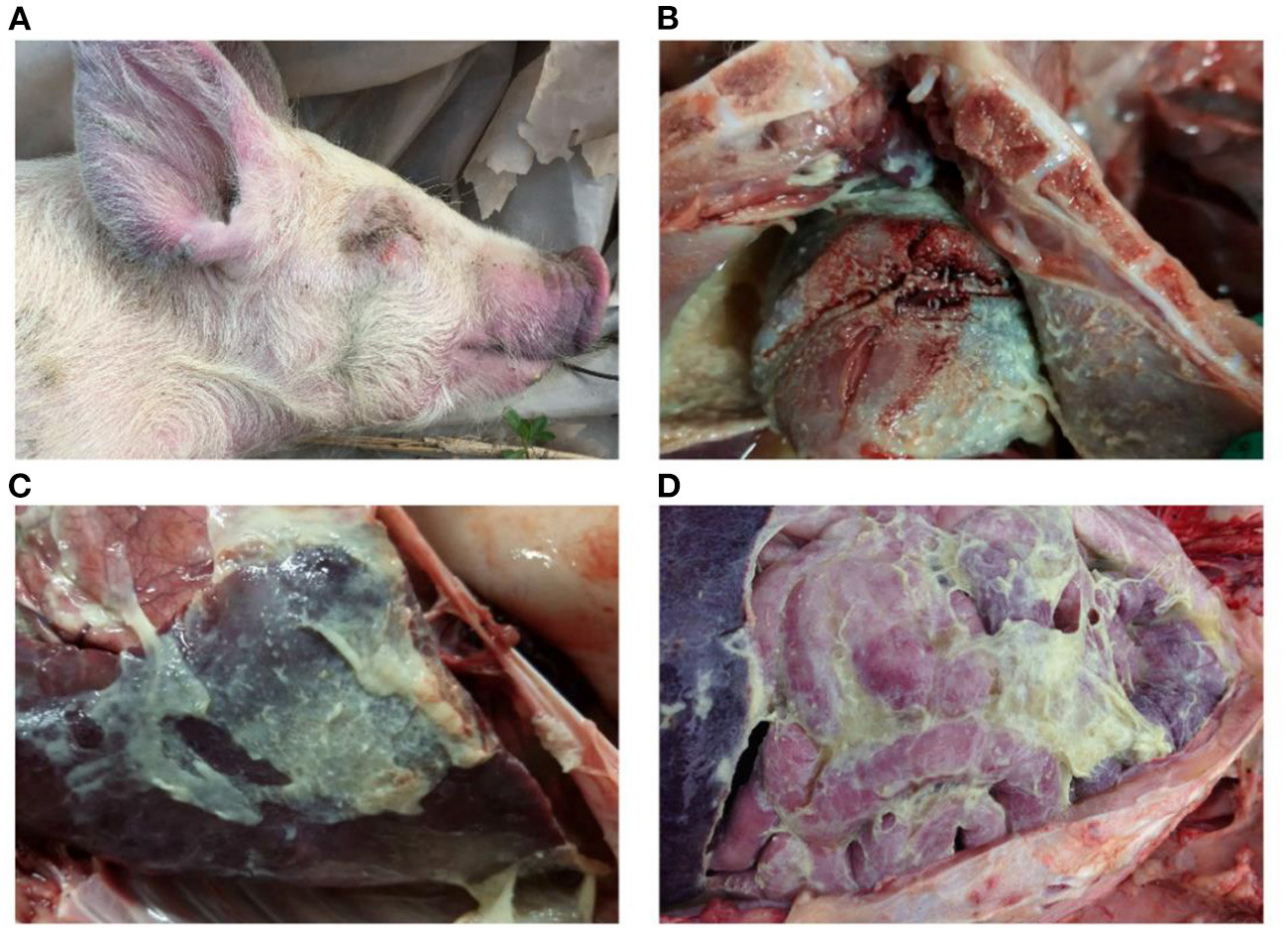
Gross lesions of Glässer's disease: **(A)** purple marks on the ears, skin around the eyes and tip of the nose of a pig that died of *H. parasuis* infection; **(B)** fibrinopurulent exudate on pericardial surface; and **(C,D)** fibrinopurulent exudate on serosal membranes in peritoneal and thoracic cavities. Photographs taken by the author.

The disease caused by *Haemophilus parasuis* (HPS), known as Glässer's disease, is characterized by fibrinous polyserositis and arthritis (Figures 1, 2). It is one of the main infectious diseases in the day-old isolated farming model of the global pig industry and causes significant economic losses (1). *H. parasuis* strains are heterogeneous in terms of phenotypic and genotypic traits. Strains have been classified into 15 serotypes, but a large proportion of isolates remain non-typable (2). Currently, vaccination is the main measure for preventing HPS infection. Commercially available inactivated bacterin vaccines are based on serovar 5, a combination of serovars 4 and 5, or a combination of serovars 1 and 6. However, all these vaccine products showed limited cross-protection against heterologous strains. There is often even failure to achieve the desired effect in protection against different isolates of the same serotype (3). Moreover, the protection against non-typable strains remains elusive. In addition, more than one strain of HPS is often present in a pig farm. For example, 4–5 strains can be isolated from a herd at a given time, and up to 16 different strains can be isolated in a single pig farm during one production cycle (4–6). This epidemiological feature also poses a great challenge for the selection of HPS vaccines in the breeding process.

Given the challenges faced by inactivated bacterial vaccines for treatment of HPS described above, the use of reverse vaccinology to develop protein vaccines against protective epitopes of the pathogen is a viable strategy. Reverse vaccinology involves computer programs to identify antigenic epitopes based on bacterial genome sequence information for vaccine development and design, avoiding the disadvantages of traditional vaccine design which is expensive and time consuming (7, 8). Moreover, with the constant updating of sequencing technologies, sequence information of bacterial genomes can be obtained at a low cost and in a short time, which also reduces the time required for vaccine design.

Hence, in this study, we used pan genome analysis to identify the core genome of HPS. Then, *in silico* prediction of B and T cell epitopes of outer membrane proteins in the core genome were performed to design a multi-epitope vaccine. An adjuvant was also ligated with to the vaccine to enhance the immunogenicity of the vaccine to obtain the final multi-epitope vaccine construct. Subsequently, the antigenicity and physicochemical properties of the vaccine construct were estimated. In addition, the secondary and tertiary structures of the construct were predicted and the interaction of the vaccine with Toll-like receptor 2 was assessed by molecular docking simulations. Finally, immune simulations were performed to confirm the immune potential of the vaccine construct and a vector was constructed for its expression in *E. coli*. The application potential of multi-epitope vaccine was preliminarily tested in mouse immunization test. Thus, in this work, a multi-epitope vaccine candidate was created using a novel vaccine design strategy based on pan-genomic analysis and reverse vaccinology techniques that will also help to accelerate the development of vaccines against other pathogens.

## Materials and methods

### Bacterial strains

In this study, we retrieved the complete genome of 105 HPS strains with rich geographical, virulent and serological diversity, which were available in March 2020 from NCBI (ftp://ftp.ncbi.nih.gov/genomes/all/). Information about the 105 strains is summarized in Supplementary Table 1.

In addition, 16 clinical strains isolated in Sichuan between 2015 and 2020 were sequenced. DNA was extracted from overnight culture using an E.Z.N.A Bacterial DNA Kit (OMEGA) following the manufacturer's guidelines and sequenced using the Min-ION MK1B platform. Raw ONT reads were corrected using Canu (9) (v1.5 https://github.com/marbl/canu, accessed on 15 October 2020) and SMARTdenovo (https://github.com/ruanjue/smartdenovo, accessed on 15 October 2020) was then used to assemble the error-corrected reads to obtain the assembled genome sequence. Lastly, Medaka (v1.0.1https://github.com/nanoporetech/medaka, accessed on 15 October 2020) and Homopolish (10) (v0.2.1 https://github.com/ythuang0522/homopolish, accessed on 15 May 2021) was used to perform multiple rounds of polishing for draft assemblies. One hundred and fifteen HPS strains were isolated from clinical cases with Glässer disease, and six strains were from the upper respiratory tract of healthy pigs.

### Pan-genomic and phylogenetic analysis

To maintain the consistency and reliability of gene prediction and annotation, a standard software tool the Prokaryotic Genome Annotation System (Prokka) pipeline (11) (v1.14.5 https://github.com/tseemann/prokka, accessed on 5 September 2021) was uniformly applied to all the 121 HPS genomes. Based on the GFF3 files produced by Prokka, the Roary program (12) (https://github.com/sanger-pathogens/Roary, accessed on 6 October 2021) was used to carry out the pan-genomic analysis to identify core and accessory genes with a minimum percentage identity of 95% between each predicted protein homolog. Then, a NJ (neighbor-joining) tree was constructed according to the core genes of HPS strains using MEGA (13) with 1,000 bootstrap replications.

### Selection of protein sequences for vaccine designing

The protein sequences of core genes and soft-core genes were extracted based on the results of the pan-genomic analysis. Then, the SignalP 5.0 server (14) was used to analyze for the presence of signal peptides (https://services.healthtech.dtu.dk/service.php?SignalP-5.0, accessed on 3 November 2021) and differentiate between secretory and non-secretory proteins. The subcellular localization of secreted proteins was further checked on Vaxign (http://www.violinet.org/vaxign/, accessed on 3 November 2021) to select outer membrane proteins as candidates for vaccine construction (15).

### Prediction of B-cell epitopes

B cells are a central component of the adaptive immune system, and they provide long-term protection against infectious pathogens by producing antibodies. In this study, linear B-cell epitopes of the candidate proteins were predicted by BepiPred-2.0 web server (16) (https://services.healthtech.dtu.dk/service.php?BepiPred-2.0, accessed on 4 November 2021).

### Prediction of cytotoxic T-lymphocyte (CTL) and helper T-lymphocyte (HTL) epitopes

The cytotoxic T-lymphocyte (CTL) epitopes from candidate protein sequences were predicted using the NetCTL 1.2 server (https://services.healthtech.dtu.dk/service.php?NetCTL-1.2, accessed on 4 November 2021). Default settings were used (threshold, 0.75) for the estimation of CTL epitopes (17). Then, the helper T cells 15-mer epitopes for candidate protein sequences were predicted by using the NetMHCII 2.3 server (https://services.healthtech.dtu.dk/service.php?NetMHCII-2.3, accessed on 4 November 2021). Seven mouse H2 class II alleles were evaluated. According to standards, the lowest consensus scores of the peptides were chosen to be the best binders and a lower percentile rank indicates higher affinity. The selection criterion was a cut-off of IC50 ≤ 50 and percentile rank <1 (18).

### Vaccine construction

A putative vaccine candidate sequence was designed by combining B-cell epitopes with high-scoring CTL epitopes and high binding affinity HTL epitopes. TLR-2 agonist, phenol soluble modulin α4 (accession no. A9JX08) protein, was preferred as an adjuvant to enhance the immunogenicity of the vaccine (19). The adjuvant was linked to the first B-cell epitope through an EAAAK linker at the N terminal of the sequence, whereas the remaining B-cell and HTL epitopes were interlinked *via* GPGPG linkers. AAY linkers were used for joining the CTLs epitopes.

### Evaluation of antigenicity, allergenicity, and physicochemical properties of the protein

In order to predict the antigenicity and allergenicity of the vaccine, VaxiJen v2.0 server (http://www.ddg-pharmfac.net/vaxijen/VaxiJen/VaxiJen.html, accessed on 5 November 2021) and AllerTOP v2.0 server (http://www.ddg-pharmfac.net/AllerTOP, accessed on 5 November 2021) were utilized, respectively (20, 21). The solubility of the designed vaccine was evaluated using the SOLpro server (22) (https://scratch.proteomics.ics.uci.edu, accessed on 5 November 2021). Furthermore, the designed vaccine was assessed for several physicochemical properties by using the ProtParam server (http://web.expasy.org/protparam/, accessed on 5 November 2021).

### Extrapolation of secondary structure of the protein

The secondary structure of the multi-epitope vaccine was predicted using PSIPRED (http://bioinf.cs.ucl.ac.uk/psipred/, accessed on 6 November 2021) server and RaptorX (http://raptorx.uchicago.edu/StructurePropertyPred/predict/, accessed on 6 November 2021) with default parameters (23, 24).

### Three-dimensional modeling and validation of the protein

Homology modeling of the final vaccine construct was performed using the Robetta server (25) (https://robetta.bakerlab.org/, accessed on 7 November 2021). Non-bond interactions between different types of atoms were analyzed using the ERRAT server (26) (http://services.mbi.ucla.edu/ERRAT/, accessed on 7 November 2021) to verify the tertiary structure. The Ramachandran plot was generated using the PROCHECK server (27) (https://servicesn.mbi.ucla.edu/PROCHECK/, accessed on 7 November 2021) to determine the relative proportion of amino acids in favored regions.

### Prediction of discontinuous B-cell Epitopes

ElliPro (http://tools.iedb.org/ellipro/, accessed on 7 November 2021) was used for prediction of B-cell discontinuous epitopes in the protein model (28).

### Molecular docking of the protein with TLR2

Toll-like receptors are sensors of the innate immune response, with TLR-2 recognizing the broadest range of PAMPs molecules and inducing significant antibacterial and antiviral responses. We used the ClusPro server (https://cluspro.bu.edu/login.php, accessed on 8 November 2021) to determine the docking of TLR-2 with the vaccine protein (29). The structural coordinates of the TLR-2 (PDB ID: 3A7C) were retrieved from the Protein Data Bank (30) (https://www.rcsb.org/, accessed on 8 November 2021). The docked structures were visualized *via* PyMOL (http://www.pymol.org, accessed on 8 November 2021) to analyze the interaction between the vaccine protein and TLR-2.

### Characterization of the construct immune profile

For analyses of the immune responses of the vaccine construct in the mouse model, the online dynamic immune simulation C-lmmSim server (https://www.iac.cnr.it/~filippo/c-immsim/index.html, accessed on 9 November 2021) was employed (31). This online server functions based on a position specific scoring matrix (PSSM) for the prediction of immunogenic epitopes and immune interactions. All default simulation parameters were used with time steps specified at 1, 90, and 180.

### *In silico* optimization and cloning of the protein

To ensure the efficient expression of the vaccine construct in *Escherichia coli* cells, we performed the reverse translation and codon optimization of the vaccine protein sequence using the Java Codon Adaptation Tool (32) (http://www.jcat.de/, accessed on 9 November 2021). *E. coli* strain K12 was selected as the expression host. During the run, the options were chosen to avoid rho-independent transcription terminators, bacterial ribosome binding sites, and restriction enzyme cleavage sites. Finally, the vaccine protein sequence was designed for cloning into a suitable host vector pET-28a(+)-MEV by employing the SnapGene software (https://www.snapgene.com/, accessed on 9 November 2021).

### Inducible expression and purification of vaccine proteins

The synthetic expression vector named pET-28a(+)-MEV is available from Chengdu YouKang Jianxing Biotechnology Co. The pET-28a(+)-MEV was transformed into *E. coli* BL21 (DE3), and positive colonies were inoculated in LB liquid medium containing kanamycin and incubated overnight in a water bath shaker at 37°C. A 10 mL aliquot of overnight culture was inoculated into 1 L of LB medium containing kanamycin, and the culture was expanded to mid-log growth (OD600 ≈ 0.6) at 37°C in a water bath shaker. IPTG was added to the culture and incubated at 37°C for 5 h. The bacteria were collected by centrifugation and ultrasonically fragmented. After sonication, the vaccine protein was purified using a Ni^2+^-NTA resin column and concentrated by desalting using an Amicon^®^ Ultra-4 10K centrifuge filter.

### Mouse immunoassay and ELISA assay

Ten 6-week-old SPFKM grade Kunming mice were randomly divided into test and control groups of five mice each. The vaccine protein was diluted to 1 μg/μL and 50 μL was mixed with an equal volume of Quick Antibody immunoadjuvant. A 100 μL aliquot of the vaccine solution was intramuscularly injected into the hind legs of mice, which were immunized again with the same dose on day 14. The control group was injected with a mixture of saline and immune adjuvant. Blood was collected from the tail vein of the mice at 21 days post-immunization and the serum was isolated and preserved. Sixteen clinical isolates of HPS were coated onto ELISA plates and the absorbance was measured at OD450 and the data recorded. Statistical analysis and visualization of the data were carried out using R 4.1.

## Results

### Nanopore sequencing and genome assembly of 16 clinical isolates of HPS

To construct a more complete pan-genome of HPS, 16 clinical isolates of HPS (HPS-1–HPS-15) were sequenced using the ONT MinION MK1B platform. Reads of each strain with a depth of about 100 × were obtained. The raw ONT reads were corrected using Canu, and SMARTdenovo was then used to assemble the error-corrected reads to obtain the assembled genome sequence. Then, the raw reads were subjected to for multiple rounds of polishing using Medaka and Homopolish to improve the quality of the draft assemblies. The data that support the findings of this study have been deposited into the CNGB Sequence Archive (CNSA) of China National GeneBank DataBase (CNGBdb) with accession number CNP0002150 (33, 34). Information about the 16 strains is summarized in Supplementary Table 2.

### Results of the pan-genomic and phylogenetic analysis

**Figure 3:**
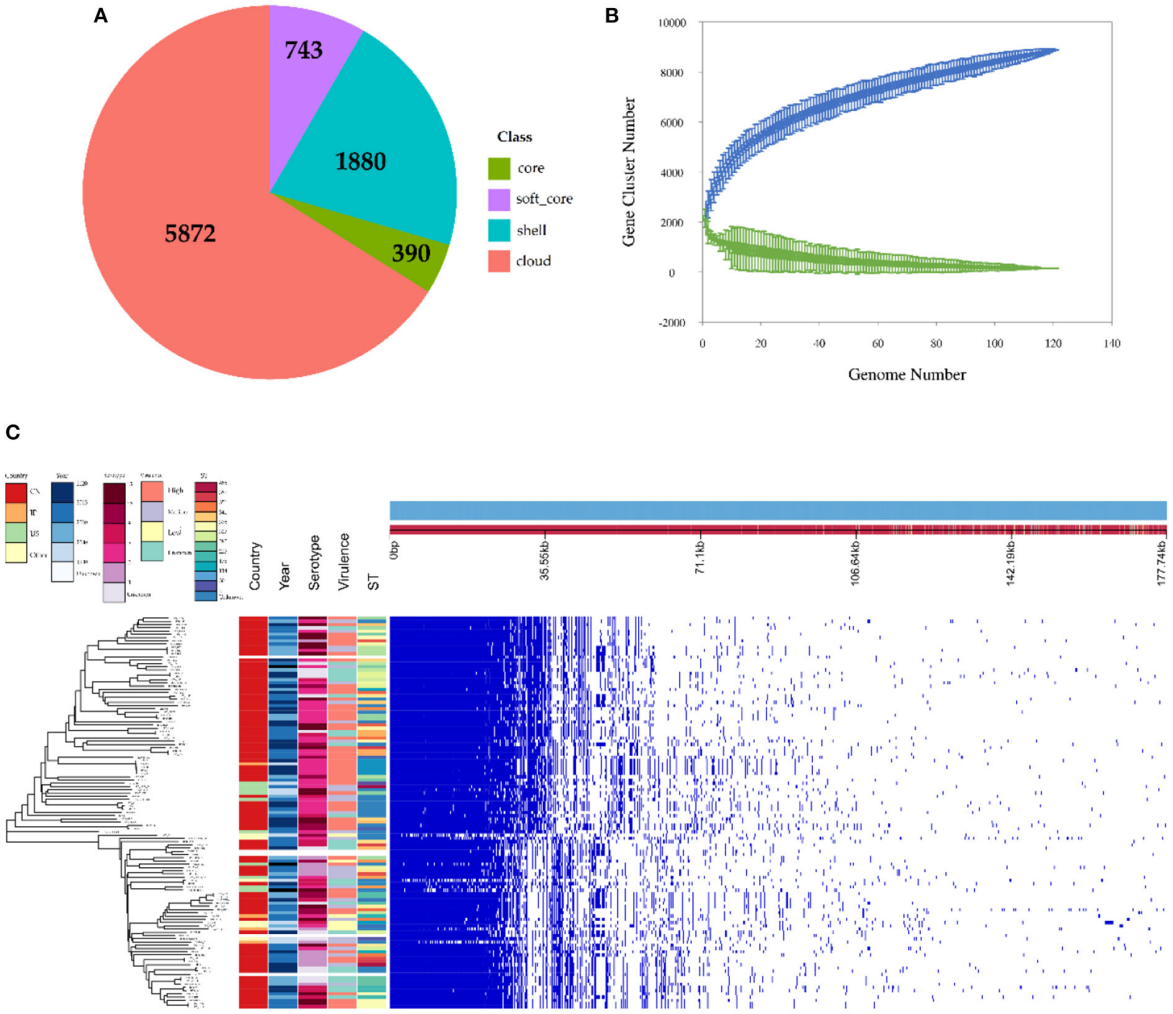
Pan-genomic and phylogenetic analysis of HPS based on 121 strains. **(A)** Core and accessory genes of HPS. Core: 119 ≤ strains ≤ 121; soft-core: 114≤strains < 119; shell: 18 ≤ strains < 114; cloud: strains < 18. **(B)** Core and pan-genome curves for HPS. The blue dots and lines indicate the size of the pan-genome for each strain combination, and the relationship between pan-genome size and genome number, respectively. The green dots and lines indicate corresponding information for the core genome. **(C)** The left tree is a neighbor-joining phylogenetic tree constructed based on the core genes and annotated with the location of isolation, time of isolation, serotype, ST type, and virulence of each strain. The right matrix plot denotes the presence and absence of every gene over all strains by blue and white, respectively.

The observed pan-genome shared by the 121 HPS strains consists of 8,885 genes including 390 core genes and 8,495 accessory genes (Figure 3A). The non-linear regression analysis showed an obvious open pan-genome, and the size of core genes approached a constant value (Figure 3B). Based on concatenated core genes, we constructed a phylogenetic tree of the HPS, which showed the rich phylogenetic diversity of HPS (Figure 3C). These results indicate that the genome of HPS is highly variable and that it can continuously obtain foreign genes to adapt to different environments. This is the main reason why conventional vaccines are less protective.

### Protein sequences for vaccine designing

Eight proteins with secreted signal peptides localized to the outer membrane were screened from the core and soft-core genes using Signal 5.0 and Vaxign (Table 1). These protein sequences were then further subjected to epitope prediction in B, T, and helper cells.

**Table 1:** Selected proteins in the core and soft-core genes of HPS.

**Protein**	**Product**
kpsD	Polysaccharide export protein
bamA	Outer membrane protein assembly factor
lptD	LPS-assembly protein
Pal	Peptidoglycan-associated lipoprotein Pal
mlaA	MlaA family lipoprotein
tama	Autotransporter assembly complex protein TamA
nlpD	Murein hydrolase activator NlpD
Gbp	Porin family protein

### Prediction of B-cell, cytotoxic T-lymphocyte (CTL), and helper T-lymphocyte (HTL) epitopes

BepiPred 2.0 server was used to select B cell epitopes with the default threshold > 0.6. Epitopes that are exposed and have a coiled structure were further selected for vaccine design (Table 2). The protein sequences were analyzed by NetCTL 1.2 server to identify the most immunodominant regions. Peptides with the highest binding affinity scores in each protein were identified as high-potential CTL epitope candidates and a total of three epitopes were screened (Table 2). The NetMHCII 2.3 web server predicted the MHC-II epitopes with the highest binding corresponding to the alleles based on the IC50 score. A total of 6 HTL epitopes were chosen for the final chimeric construct (Table 2).

**Table 2:** Predicted B-cell, CTL, and HTL epitopes for the design of the vaccine protein.

	**Protein**	**Epitope sequence**
B-cell epitopes	KpsD	APRAVKASDNIGLEQQIKR
	bamA	AQREFNRELYVQSMKFPIDNDLNVYKKI
	LptD	QGNIGVRNPKYLGL
	pal	NAGQTFGGMSAQDL
	gbp	NINNAPKAGTTYGNWHAPKRES
HTL epitopes	tama	YLADWRLGY
	LptD	ETKLYTTYY
	LptD	YVENDSTTY
	bamA	TTPGSDNKY
	mlaA	ATPWSITKY
	nlpD	TAANNTPNY
CTL epitopes	gbp	DPILTKWASAIVAKK
	gbp	ILTKWASAIVAKKNQ
	tama	AGIGVRWASPIGAVK

### Vaccine construction

**Figure 4:**
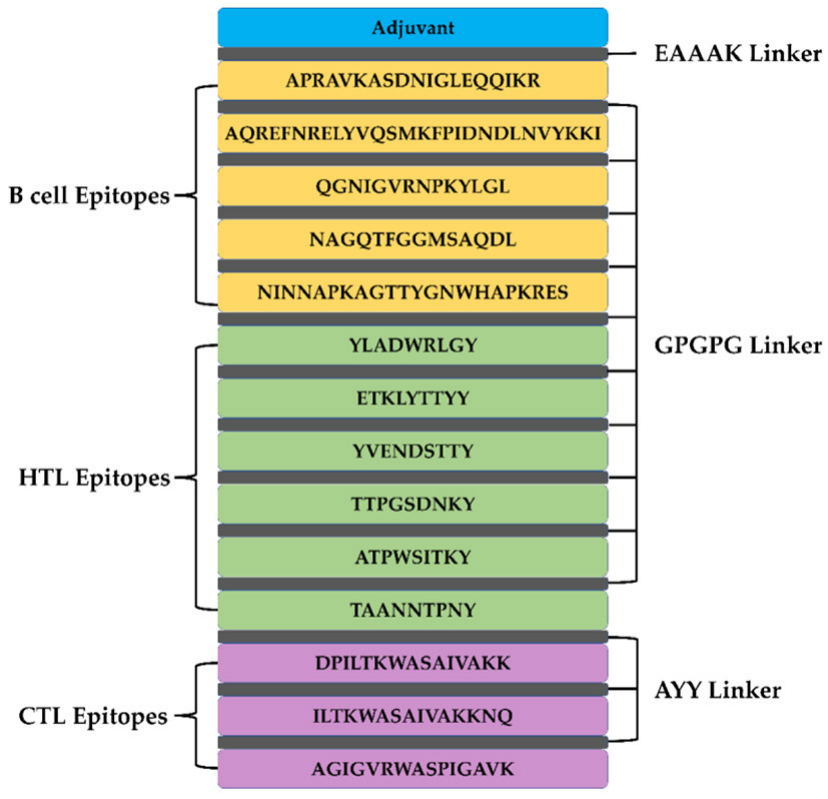
The structural arrangement of B- and T-cell epitopes along with linkers and adjuvant for the final multi-epitope vaccine construct.

A total of 5 B cell epitopes, six HTL epitopes and three HTL epitopes were used to construct multi-epitope vaccine chimeras. The B cell and HTL epitopes were linked together by GPGPG linkers, while AY linkers were used to link the CTL epitopes. Phenolic soluble modulin α4 (UniProt Id: A9JX08), a TLR-2 agonist, was added as an adjuvant at the N-terminal end with an EAAAK linker to enhance the immunogenicity of the vaccine. The final chimeric construct constituted 280 amino acids (Figure 4).

### Antigenicity, allergenicity, and physicochemical properties of the vaccine protein

VaxiJen 2.0 web server was used to predict the antigenicity of the vaccine design attached with an adjuvant, as 0.7756 with the bacterial model by opting for a threshold of 0.4. The antigenicity of the vaccine candidate was also checked without including the adjuvant part for which VaxiJen gave scores of 0.7114 in a model of bacteria. AllerTOP v.2 and AllergenFP online servers predicted the vaccine sequence to be non-allergenic in nature in the presence and absence of the adjuvant.

### Solubility and physicochemical properties of the vaccine protein

The SOLpro server of the Scratch protein prediction tool predicted a solubility probability of 0.887834 for this vaccine protein. ExPASy ProtParam was used to predict the molecular weight (MW) of the vaccine protein as 29.4 kDa. The pI (theoretical isoelectric point value) of the protein was calculated as 9.65. The estimated half-life of the protein in mammals, yeast, and E. coli was estimated as 30 h, >20 h, and >10 h, respectively. In addition, the protein was predicted to have an instability index (II) of 24.99 by ProtParam, classifying it as stable since a value of >40 indicates instability. The values of 70.03 and −0.438 for the aliphatic index and GRAVY (grand average of hydropathicity) reflect the high thermostability and hydrophilic nature, respectively.

### Secondary structure of the vaccine protein

**Figure 5:**
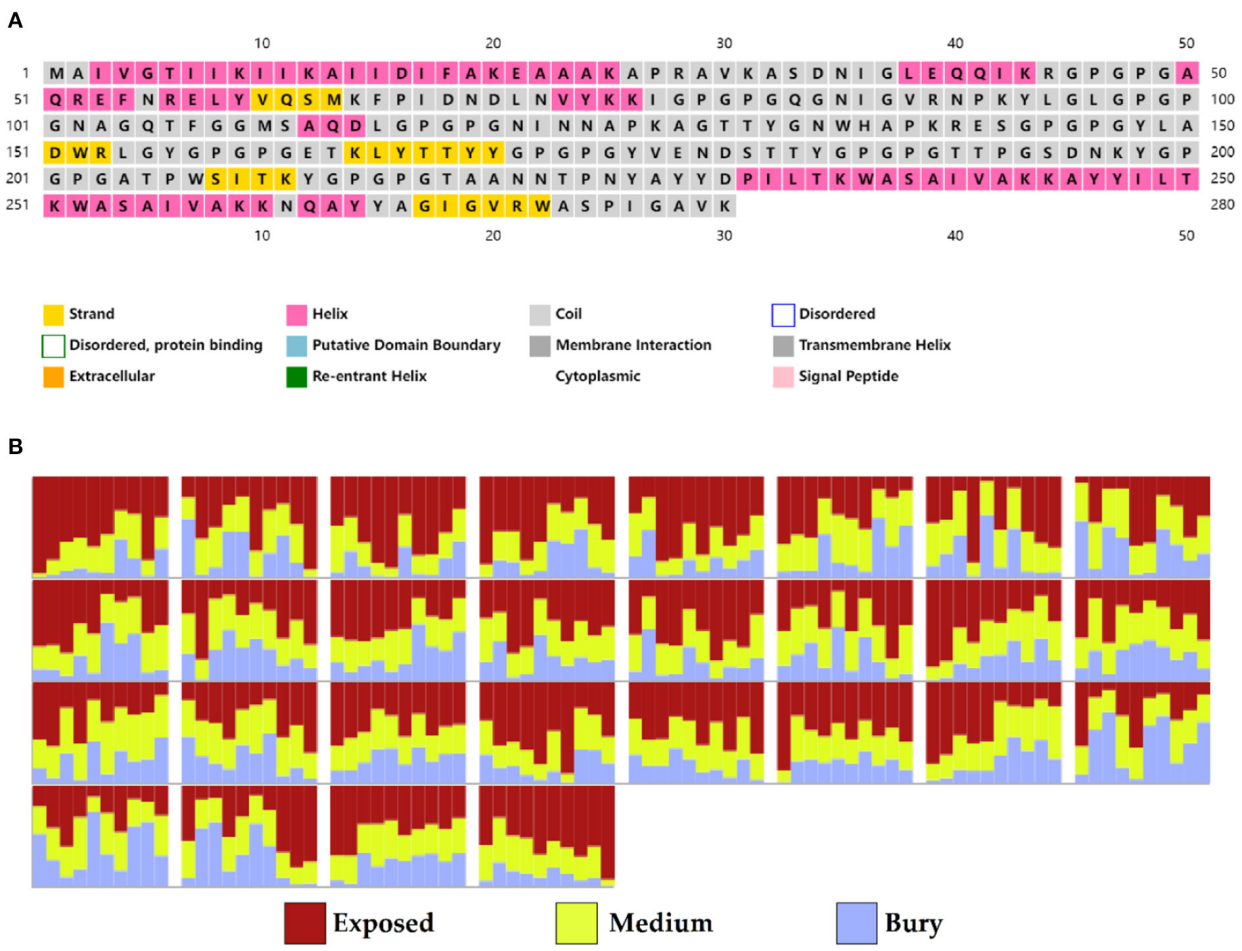
Secondary structure prediction of the vaccine protein sequence by using PSIPRED and RaptorX server showing **(A)** secondary structural elements and **(B)** solvent accessibility according to three states.

Based on PSIPRED and RaptorX server results, the protein vaccine consists of 22% helix (H), 3% beta strand (E), and 73% coil (C) secondary structural elements (Figure 5A). Based on the accessibility of amino acid residues, 62% residues were predicted to be exposed, 18% medium exposed, and 18% were predicted to be buried (Figure 5B). In total, only 2% of residues were found to be localized in disordered structural domains.

### Three-dimensional modeling and validation of the vaccine protein

**Figure 6:**
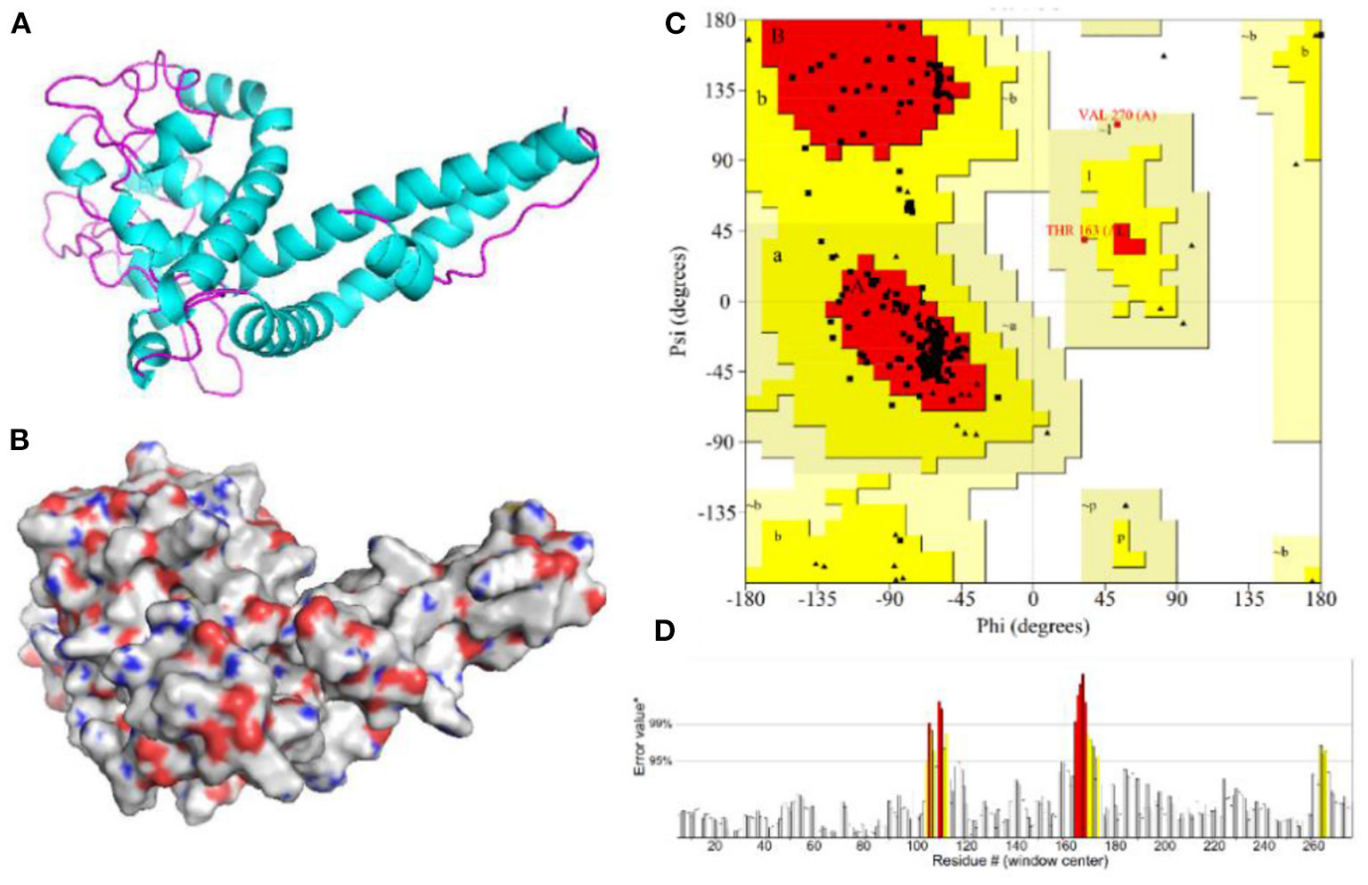
Homology modeling and validation of the three-dimensional structure of vaccine constructs. **(A,B)** 3D models of vaccine constructs generated by homology modeling on Robetta and visualized by PyMOL software. **(C)** Ramachandran plot analysis shows 100% of residues in the allowed region. **(D)** The overall quality factor score for the model was 92.647%.

The 3D model of the vaccine protein was generated by Robetta server (Figure 6) and was visualized using PyMOL software. Ramachandran plot analysis showed that 100% of the residues were in the allowed regions and 91.1% of the residues were in the most favored regions. The overall quality factor score generated by the ERRAT2 was 92.647%. The above results for the 3D model demonstrate that the vaccine construct has a stable chemical structure.

### Prediction of discontinuous B-cell epitopes

**Figure 7:**
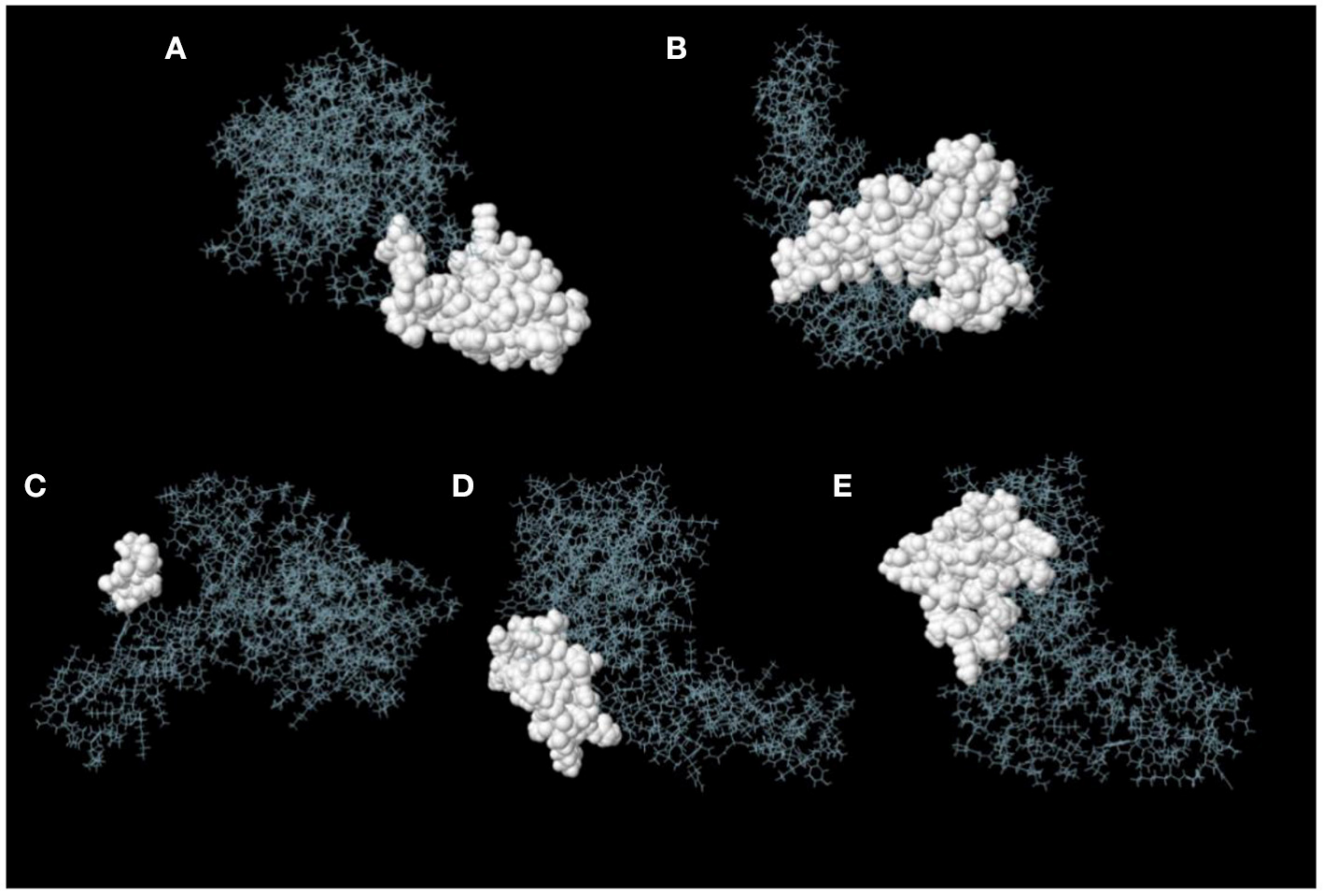
Discontinuous B-cell epitopes of vaccine constructs predicted by ElliPro. **(A–E)** The epitopes are represented by a white surface. The gray sticks correspond to the remainder of the protein.

ElliPro server was used to predict discontinuous B-cell epitopes for the vaccine constructs. The results showed that the vaccine constructs had five discontinuous B-cell epitopes that ranged in size from 7 to 38 residues, with scores ranging from 0.588 to 0.822 (Figure 7 and Table 3).

**Table 3:** Discontinuous B-cell epitopes of vaccine constructs predicted by ElliPro.

**No**.	**Residues**	**Number of**	**Score**
		**residues**	
A	A:M1, A:A2, A:I3, A:V4, A:G5, A:T6, A:I7, A:I8, A:K9, A:I10, A:I11, A:K12, A:A13, A:I14, A:D16, A:I17, A:T250, A:K251, A:A253, A:S254, A:A255, A:I256, A:V257, A:A258, A:K259, A:K260, A:N261, A:Q262, A:A263, A:Y264, A:Y265, A:A266, A:G267, A:I268, A:G269, A:V270, A:R271, A:W272	38	0.822
B	A:P138, A:K139, A:R140, A:E141, A:S142, A:G143, A:P144, A:G145, A:P146, A:G147, A:Y156, A:G157, A:P158, A:G159, A:P160, A:G161, A:E162, A:T163, A:K164, A:Y166, A:Y169, A:Y170, A:G171, A:P172, A:G173, A:P174, A:G175, A:Y176, A:V177, A:E178, A:N179, A:D180, A:S181, A:T182, A:T183, A:Y184, A:G185, A:P186, A:G187, A:P188, A:G189, A:T190, A:T191, A:P192, A:G193, A:S194, A:D195, A:Y198	48	0.678
C	A:A273, A:S274, A:P275, A:G277, A:A278, A:V279, A:K280	7	0.664
D	A:N35, A:I36, A:L38, A:E39, A:Q40, A:Q41, A:I42, A:K43, A:R44, A:G45, A:P46, A:G47, A:P48, A:G49, A:A50, A:Q51, A:R52, A:E53, A:G116, A:I122, A:N123, A:N124, A:A125, A:P126, A:K127, A:G129, A:T130, A:T131, A:Y132	29	0.664
E	A:L71, A:Y74, A:K75, A:I77, A:G78, A:P79, A:G80, A:P81, A:G82, A:Q83, A:G84, A:N85, A:I86, A:G87, A:V88, A:R89, A:N90, A:P91, A:K92, A:Y93, A:L96, A:G97, A:P98, A:G99, A:P100, A:G101, A:N102, A:A103, A:G104, A:Q105, A:G109, A:G215, A:P216, A:G217, A:T218	35	0.588

### Molecular docking of the vaccine protein with TLR2

**Figure 8:**
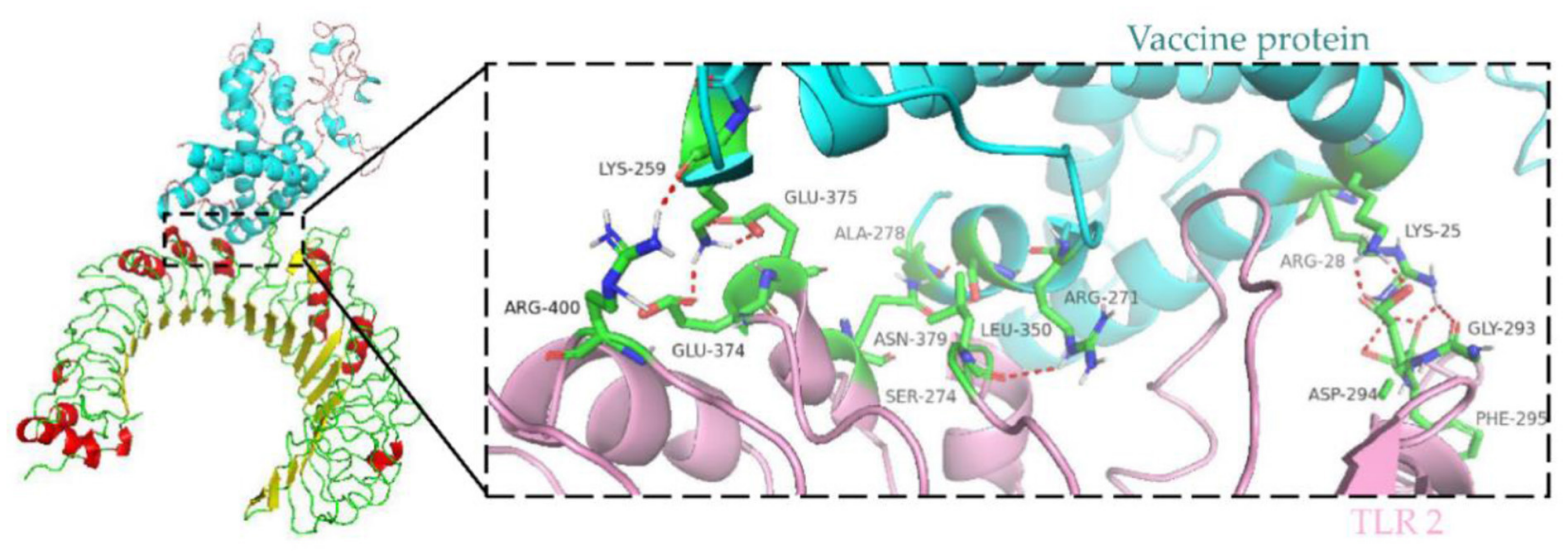
Molecular docking of subunit vaccine with TLR2. Docking complex of vaccine protein and TLR2, with the vaccine protein colored sky blue and the A chain of TLR2 colored light pink. Residues with H-bond interactions are represented in the sticks model and the remaining residues are represented in the cartoon model. Hydrogen bonds are represented as red dashed lines.

Immune cells recognize evolutionarily conserved pathogen patterns in a targeted manner and respond by expressing Toll-like receptors. Within the TLR family, TLR2 has a broad recognition spectrum, recognizing lipoproteins, lipopolysaccharides, peptidoglycans and other signals indicating danger (35). Therefore, we used the ClusPro server to determine the docking of TLR 2 with the vaccine protein. The central energy between ligand receptor is −820.2, and the lowest energy of the docking complex is −857.6. The vaccine protein has multiple hydrogen-bonded interactions with TLR2 and exhibits a high binding affinity. The residues of the vaccine-TLR2 complex showing H-bond interactions are LYS25–ASP294, ARG28–GLY293, ARG28–PHE295, ARG271–LEU350, SER274–ASN379, ALA278–ASN379, LYS259–ARG400, LYS259–GLU374, and LYS259–GLU375 with a distance of 2.1, 1.8, 1.8, 2.2, 2.4, 1.6, 2.1, 1.9, and 1.9Å, respectively (Figure 8).

### Immune simulation of the vaccine construct

**Figure 9:**
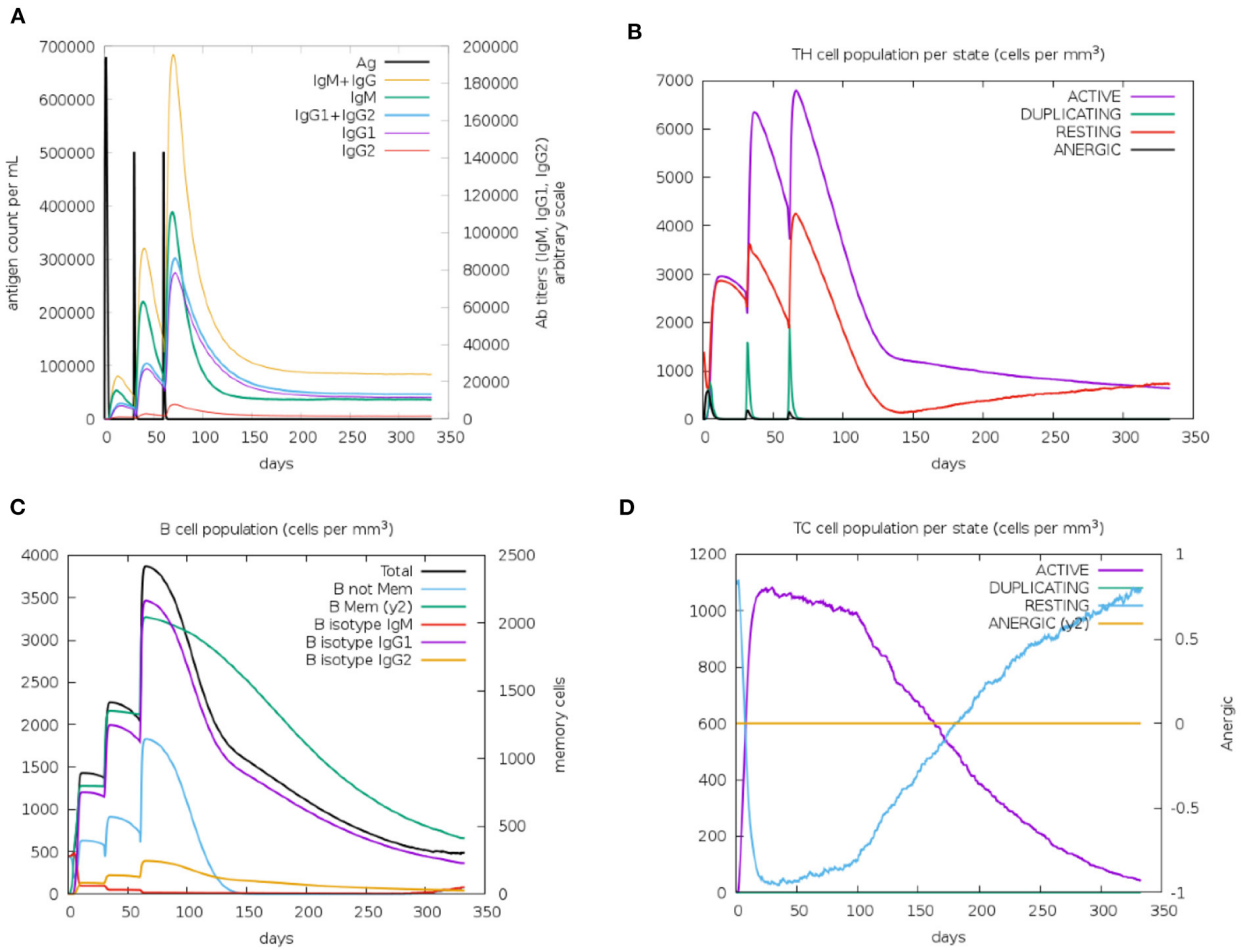
Simulated immunity of vaccine constructs using C-ImmSim server showing **(A)** an increase in immunoglobulins following vaccination **(B,D)**, that the population of cytotoxic T-lymphocytes and helper T-lymphocytes increases after vaccination and remains higher during the entire exposure time, and **(C)** an increase in B-cell populations after vaccination, producing high levels of memory immunoglobulins.

Immunological characterization of the designed vaccine construct was analyzed using the C-ImmSim server. The C-ImmSim server immune simulation outcomes confirmed consistency with real immune reactions. As shown in Figure 9A, IgM production was recorded in the first injection of the vaccine construct and increased levels of IgM+IgG and IgG1+IgG2 were observed after the second and third immunizations. High levels of B-cell populations were observed after multiple injections of the vaccine constructs, indicating the formation of immune memory (Figure 9C). In addition, an increase in cytotoxicity and helper T-lymphocytes was observed following vaccination, thus indicating activation of cell-mediated immune responses (Figures 9B,D). The above results demonstrate the ability of the vaccine construct to induce an effective immune response to clear the antigen.

### Codon optimization and *in silico* cloning

**Figure 10:**
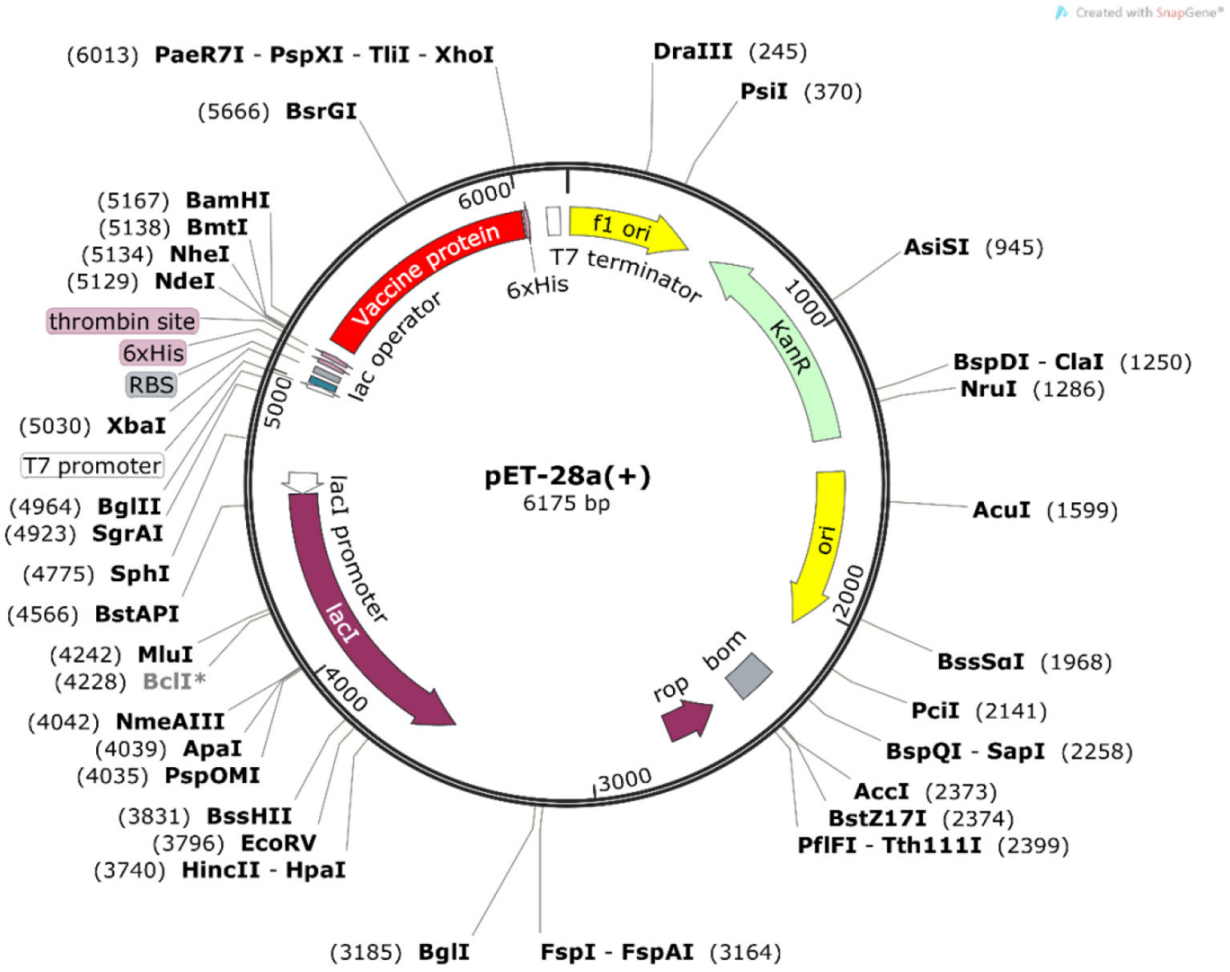
*In silico* cloning of the vaccine construct. The codon-optimized DNA sequence of the vaccine construct (in red) was cloned between the BamH I and Xho I restriction sites of the pET-28a(+) expression vector (shown in black circles).

The Java Codon Adaptation Tool was used for codon optimization. The vaccine protein sequence was reverse translated for optimal expression in E. coli (strain K12). The optimized DNA sequence had a codon adaptation index (CAI) value of 1 and a GC content of 54.05%, indicating that the designed vaccine is theoretically stably expressed in the selected microbial hosts. In addition, the DNA sequence for cloning into the E. coli vector pET-28a(+) was designed using the SnapGene software for recombinant plasmid construction (Figure 10).

### Inducible expression and purification of vaccine proteins

**Figure 11:**
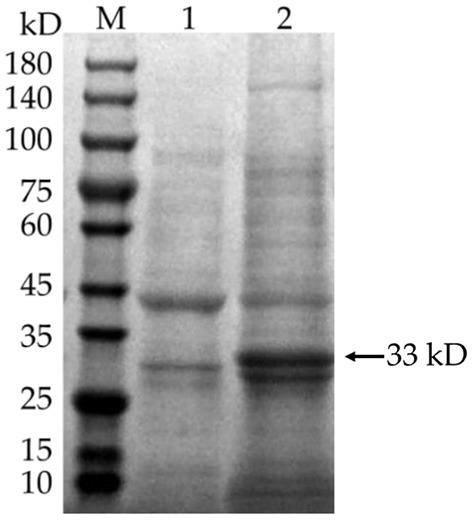
Induced expression of the vaccine protein. M: protein molecular quality standard (10–180 kD); 1: pET-28a(+)-MEV recombinant bacteria, uninduced; 2: pET-28a(+)-MEV recombinant bacteria, induced. Full-length gels are presented in Supplementary Image 1.

IPTG was added to E. coli BL21 (DE3) to induce the expression of pET-28a(+)-MEV, and the product was examined by SDS-PAGE gel electrophoresis. The results showed that the induced pET-28a(+)-MEV recombinant produced a specific protein band around 33 kD as expected (Figure 11), whereas the uninduced sample did not show such a band. The sonicated lysates were purified using a Ni^2+^-NTA resin column, the eluate was collected and concentrated using an Amicon^®^ Ultra-4 10K centrifuge filter, and the final vaccine protein was stored in 10% glycerol at −80°C.

### ELISA of 16 HPS clinical isolates

**Figure 12:**
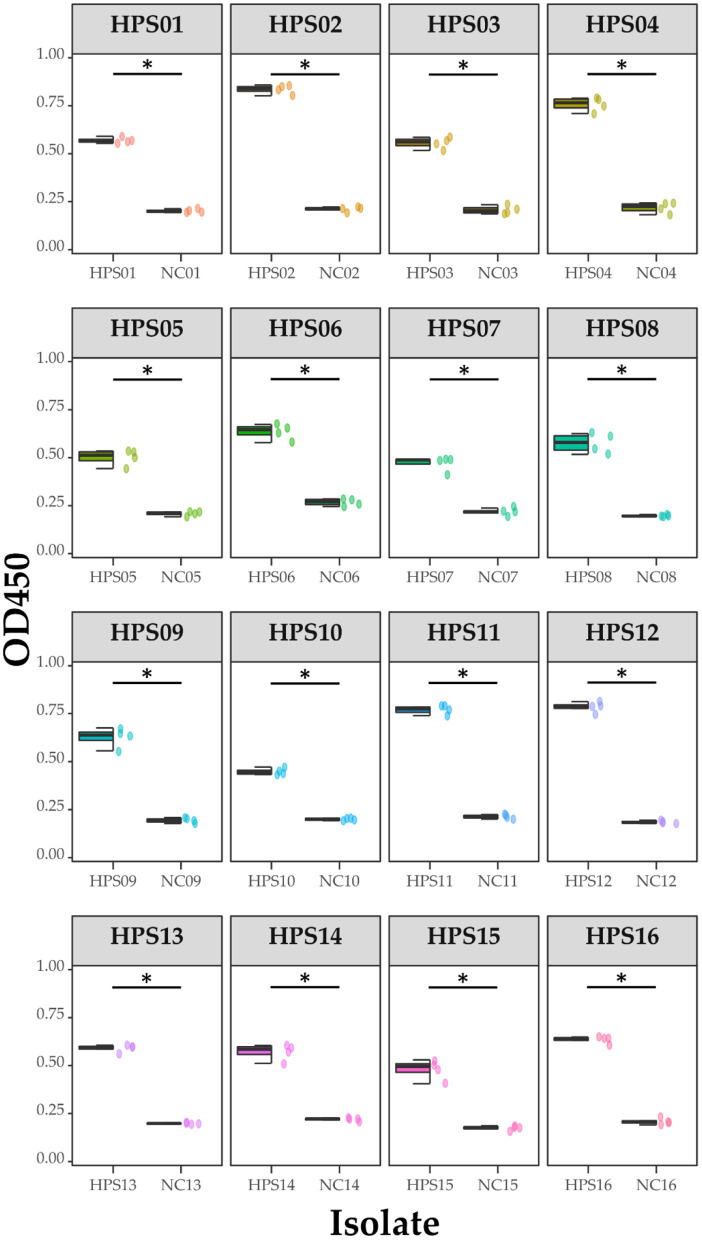
ELISA results for each isolate. Data are presented using box and scatter plots; NC is negative serum control; * indicates statistically significant difference.

Pure cultures of each of the 16 HPS clinical isolates were used to coat enzyme-labeled plates at the same concentration and volume, and ELISA assays were performed using mouse polyclonal antibodies obtained by immunization with a multi-epitope vaccine, with a negative serum control. The results are shown in Table 4 and Figure 12: the mouse polyclonal antibodies obtained were able to bind to all 16 HPS clinical isolates, including seven serotype 5, two serotype 10, one serotype 1, two serotype 7, and four non-typable strains, and a one-tailed heteroscedasticity t-test was performed on the OD450 values of each isolate against the corresponding negative serum OD450 values, with all the p-values being less than the test level (α = 0.05), indicating the differences were significant. These results indicate that the antibodies obtained from mice immunized with the multi-epitope vaccine designed in this study were able to bind to different serotyped or non-typable HPS isolates.

**Table 4:** ELISA results for each isolate.

**Isolates**	**Isolate**	**Mean ±**	**Negative**	**Mean ±**	***p*-**
		**SD**	**serum**	**SD**	**value**
			**OD450**	
	**OD450**				
HPS 01	0.563	0.57 ± 0.0158	0.214	0.202 ± 0.009	1.53E-07
	0.591		0.202		
	0.57		0.196		
	0.554		0.194		
HPS 02	0.847	0.835 ± 0.0243	0.213	0.211 ± 0.0129	1.49E-07
	0.802		0.192		
	0.858		0.222		
	0.833		0.215		
HPS 03	0.517	0.556 ± 0.0295	0.234	0.207 ± 0.0213	1.60E-06
	0.571		0.193		
	0.585		0.213		
	0.551		0.187		
HPS 04	0.79	0.757 ± 0.037	0.237	0.219 ± 0.0278	4.56E-07
	0.709		0.182		
	0.782		0.243		
	0.748		0.212		
HPS 05	0.535	0.501 ± 0.0419	0.216	0.209 ± 0.0118	0.000203
	0.497		0.208		
	0.443		0.192		
	0.528		0.218		
HPS 06	0.673	0.635 ± 0.0416	0.281	0.268 ± 0.0189	3.16E-05
	0.657		0.285		
	0.633		0.245		
	0.578		0.259		
HPS 07	0.485	0.469 ± 0.0389	0.194	0.217 ± 0.018	0.000109
	0.411		0.218		
	0.491		0.218		
	0.49		0.238		
HPS 08	0.626	0.575 ± 0.0516	0.192	0.197 ± 0.0053	0.000312
	0.547		0.197		
	0.61		0.194		
	0.517		0.204		
HPS 09	0.646	0.627 ± 0.0507	0.199	0.194 ± 0.0129	0.000116
	0.675		0.208		
	0.63		0.178		
	0.556		0.189		
HPS 10	0.432	0.447 ± 0.018	0.206	0.2 ± 0.0066	1.06E-05
	0.436		0.192		
	0.472		0.196		
	0.449		0.204		
HPS 11	0.784	0.768 ± 0.021	0.224	0.213 ± 0.0103	2.18E-07
	0.74		0.201		
	0.763		0.209		
	0.784		0.219		
HPS 12	0.79	0.784 ± 0.0279	0.18	0.185 ± 0.0076	4.49E-06
	0.746		0.195		
	0.788		0.178		
	0.813		0.186		
HPS 13	0.606	0.59 ± 0.0202	0.203	0.198 ± 0.0041	1.04E-05
	0.597		0.198		
	0.595		0.193		
	0.56		0.198		
HPS 14	0.595	0.571 ± 0.0422	0.221	0.22 ± 0.0089	0.000153
	0.605		0.207		
	0.573		0.222		
	0.511		0.228		
HPS 15	0.53	0.481 ± 0.0538	0.176	0.175 ± 0.0126	0.000482
	0.485		0.187		
	0.503		0.157		
	0.405		0.178		
HPS 16	0.64	0.634 ± 0.0192	0.206	0.209 ± 0.0168	3.04E-08
	0.606		0.232		
	0.642		0.204		
	0.649		0.192		

## Discussion

Haemophilus parasuis mainly causes Glässer's disease, characterized by fibrinous polyserositis and arthritis in nursery pigs and affecting growing pigs and sows. Vaccination is an effective measure to prevent mortality (36, 37). However, the available commercial vaccines only provide protection against a limited number of serotypes. Although autogenous vaccines are highly effective in protecting susceptible pigs, the strains used to prepare the vaccines are not isolated until after a disease outbreak (38). Furthermore, for the treatment of HPS infection, the timing of antibiotic administration is critical, and effective treatment should be administered before fibrinous inflammation develops. However, due to the difficulty of early diagnosis, the sick pigs have usually developed severe fibrinous inflammation by the time of diagnosis. Even if the pathogen has been killed by antibiotics, the pigs may still die due to the inflammation-induced cytokine storm and irreversible histopathological damage (39), and conventional antibiotic treatment is often ineffective. Therefore, this study aims to design a multi-epitope vaccine that is expected to provide broad protection against HPS infection in pig farms (all serotypes and non-typable strains). Pan-genomic analysis was used to identify the core genome of HPS, software programs and databases were used to predict the antigenic epitopes of outer membrane proteins in the core genome, and reverse vaccinology techniques were used to design the final multi-epitope vaccine, reducing traditional laboratory-based experimental practices.

Herein, we first sequenced the genomes of 16 HPS strains using Oxford nanopore sequencing technology (ONT) and obtained complete assemblies of high quality. Compared with second-generation sequencing platforms, the ONT platform has the advantages of lower cost, faster sequencing speed, longer read length, and greater ease of operation (40). Subsequently, the 16 HPS genomes obtained together with the 105 NCBI-indexed genomes were used to construct the HPS pangenome using Roary software. The analysis results showed that HPS has an open pan-genome. The number of core genes represents only 14% of the CDS of an individual genome, and the rest is highly variable. The large variation in the genomes of different isolates of HPS is the main reason for the lack of cross-protection of existing vaccines. It is also worth clarifying that not only virulent strains but also non-virulent strains were used to construct the pan-genome. The reason for this is that non-virulent strains have the potential to be converted into virulent strains. For example, serovar 7 strains were considered to all be non-virulent, but some serovar 7 strains have been isolated from systemic lesions of Glässer's disease, and disease has been reproduced with one of them (41, 42).

The following eight outer membrane proteins were identified from core and soft core genes by signal peptide prediction and subcellular localization: (1) Podoconjugate export protein kpsD—this protein is mainly involved in translocation of podoconjugates from the site of synthesis to the cell surface (43). (2) Outer membrane protein assembler bamA—bamA is mainly involved in catalyzing the assembly of bacterial transmembrane proteins and could be a potential vaccine candidate for the prevention of Escherichia coli and Salmonella infections (44, 45). (3) Lipopolysaccharide assembly protein lptD—this protein mediates lipopolysaccharide transport and lptD of Vibrio Parahemolyticus is highly immunogenic, providing 100% protection against Vibrio infection in mice and is a potential vaccine antigen (46). (4) Peptidoglycan-associated lipoprotein Pal—this protein interacts with Tol Pal is a natural TLR2 agonist and binds tightly to LPS, which is released into the bloodstream during infection causing sepsis (47, 48). In addition, Pal from Legionella pneumophila, Haemophilus influenzae, and Campylobacter jejuni was shown to be highly immunogenic and capable of inducing early innate and adaptive immune responses (49–51). (5) Lipoprotein mlaA—this protein, which belongs to the same class of lipoproteins as Pal, is also involved in maintaining the lipid asymmetry of the outer membrane of Gram-negative bacteria, forming an osmotic barrier to prevent the entry of toxic molecules (e.g., antibiotics, disinfectants, etc.). (6) The autotransport assembly complex protein TamA—studies suggest that this protein may be involved as a substrate for secretion to facilitate the secretion of autotransport proteins rather than in an autotransport system for pathogen colonization in the host (52). (7) Urea hydrolase-activating protein nlpD—this protein is also a lipoprotein, and in Cronobacter sakazakii, nlpD responds to acid stress to resist phagocytosis by maintaining membrane integrity. In addition, nlpD may also be involved in the regulation of iron uptake and the activity of the bis-arginine system (53, 54). (8) Pore protein gbp—pore proteins are abundantly present on the surface of bacteria as a sieving barrier and play an important role in host-bacteria interactions, making them potential vaccine candidate antigens and therapeutic targets (55). Then, B cell epitopes (n = 5), CTL epitopes (n = 3), and HTL epitopes (n = 6) were identified from these outer membrane proteins for use in the construction of the vaccine. To improve the stability of the vaccine structure, epitopes were linked together using the EAAAK linker and the GPGPG linker (56, 57). The multi-epitope vaccine was then linked to the phenol-soluble modulin α4 protein (a TLR 2 agonist) selected as an adjuvant using an AYY linker to enhance the immunogenicity of the vaccine (19). The vaccine construct was subsequently tested for antigenicity and allergenicity and was shown to be antigenic and non-allergenic with or without linking to the adjuvant; a higher antigenicity score was predicted for coupling with the adjuvant.

The final vaccine construct containing B-cell epitopes, CTL epitopes, and HTL epitopes as well as linkers and adjuvant is 280 amino acid residues and has a molecular weight of 29.38 kD. The theoretical pI of this vaccine protein was 9.65, indicating the basic nature of the protein. The instability index of the protein is 24.99, whereby a value of <40 indicates that the vaccine construct will be stable whenever expressed (58). In addition, other indicators demonstrated the high thermostability, hydrophilic nature and solubility of the vaccine construct.

The secondary structure prediction of the vaccine constructs showed that only 2% of the amino acid residues were disordered, confirming the stability of the constructs. The construct is mainly composed of coils (73%), which will facilitate antibody production. Moreover, the vaccine construct was modeled and validated, and the interaction of the vaccine construct with TLR2 was investigated using molecular docking simulations to elucidate an effective immune response. The validation of the 3D structure showed that all the residues were in the allowed regions, and over 90% were present in favorable regions, confirming that we obtained a high-quality structural model. Furthermore, the construct had a high binding affinity to TLR2, indicating that it has the potential to stimulate the generation of an immune response. According to the immune simulation results of the C-ImmSim server, a high level of memory B cell formation and antibody production, as well as an increased and prolonged maintenance of cytotoxic and helper T-lymphocytes could be observed after multiple immunizations, thus creating a humoral and cellular immune response that will help prevent infections. In addition, codon optimization was performed after reverse translation of the vaccine protein. The GC and CAI values predicted for the vaccine protein were 54.05% and 1 respectively, indicating that the protein can be expressed in large quantities in E. coli.

The vaccine protein was reverse-translated and codon-optimized, and the E. coli expression vector pET-28a(+)-MEV was constructed and transferred to E. coli BL21 (DE3) for induction of expression, and the target vaccine protein was purified. Polyclonal antibodies obtained from mice immunized with the vaccine protein were able to bind to different serotypes or non-typable HPS isolates, preliminary indicating that the vaccine protein is a promising vaccine candidate. The multi-epitope vaccine designed in this study also has multiple clinical use strategies based on the infection and epidemiological characteristics of HPS—For example, in the direct immunization of piglets against HPS infection. In addition, because the virulent strain of HPS can colonize the respiratory tract of piglets under the protection of maternal antibodies and thus stimulate the piglet organism to produce an immune response to prevent morbidity and mortality, this process is limited to the strain to which the sow has been exposed. Therefore, an alternative immunization strategy that may be more effective is the use of multi-epitope vaccines to immunize reserve or pregnant sows to stimulate the production of antibodies against various serotypes or non-typable HPS, with the piglets being protected by maternal antibodies exhibiting a wide range of reactivities and colonized by different strains of HPS virulence, thus producing an immune response to prevent infection.

## Conclusion

The development of a new vaccine is necessary to address the complex epidemiological situation of Haemophilus parasuis and to solve the problems associated with existing vaccines. In this study, we utilized pan-genomic analysis with reverse vaccine technology to construct a vaccine with the potential to prevent infection by all serotypes as well as non-typable of Haemophilus parasuis, and this process that avoids the high cost and time-consuming drawbacks of traditional vaccine development. The vaccine construct had multiple B and T cell epitopes and exhibited high antigenicity, non-toxicity and non-allergenicity. In addition, immune simulation results showed that the vaccine activated high levels of humoral and cellular immune responses. The antibodies obtained from mice immunized with the multi-epitope vaccine were able to bind to different serotyped or untypable HPS isolates. In conclusion, the vaccine designed in this study is a promising candidate for the control of Haemophilus parasuis.

## Data availability statement

The original contributions presented in the study are included in the article/Supplementary material, further inquiries can be directed to the corresponding author.

## Ethics statement

The animal study was reviewed and approved by the Sichuan Provincial Laboratory Animal Management Committee.

## Author contributions

MP and TT: methodology. MP and MR: software. PZ and TT: validation. PZ and YL: data curation. MP: writing-original draft preparation. TT and YW: writing-review and editing. XY and ZY: project administration. All authors have read and agreed to the published version of the manuscript.
