# Supplementary figures and images for: Design of a multi-epitope vaccine against Haemophilus parasuis based on pan-genome and immunoinformatics approaches
Source: Front Vet Sci. 2022 Dec 29;9:1053198. doi: 10.3389/fvets.2022.1053198 (PMC9835091; doi:10.3389/fvets.2022.1053198)

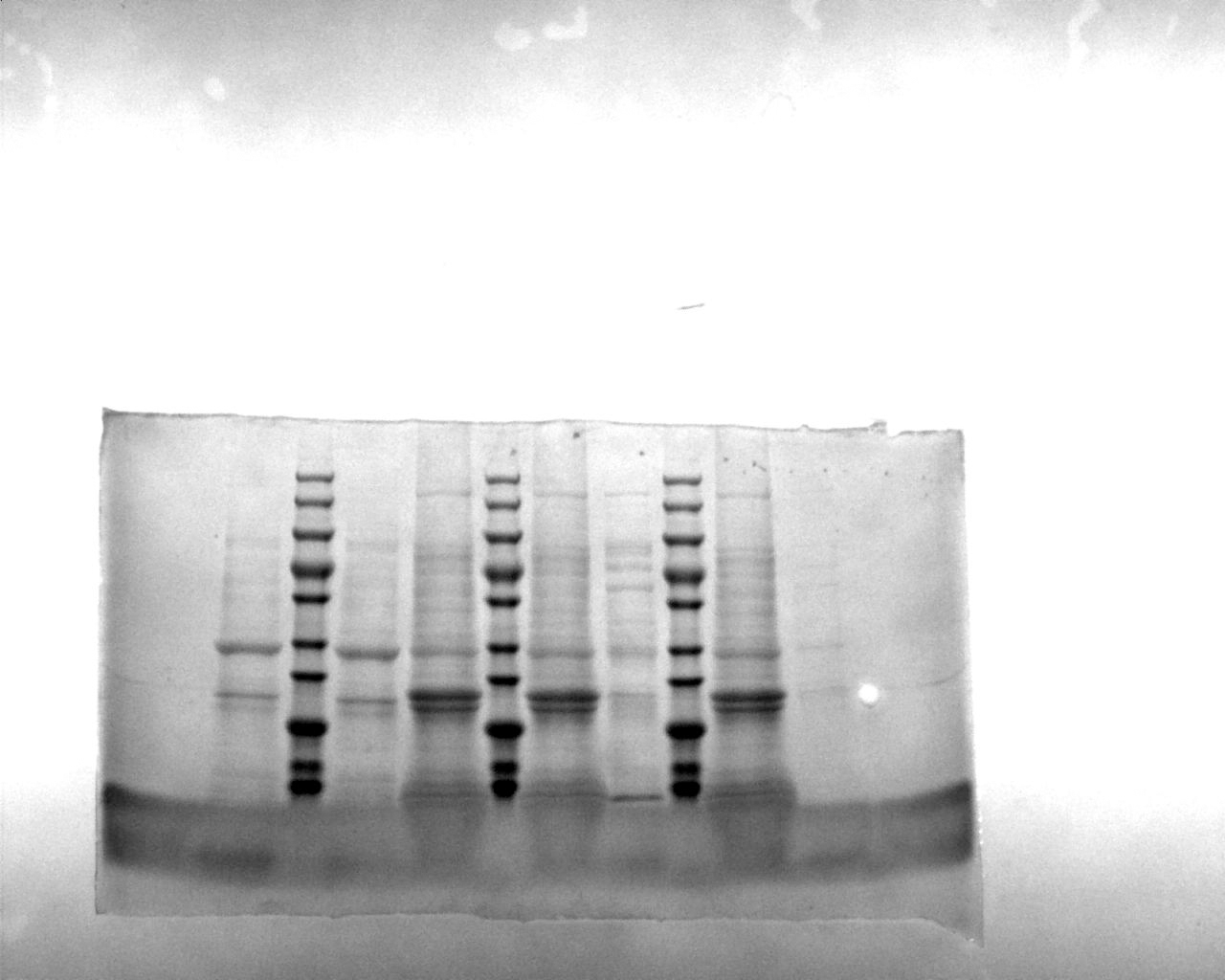

Supplement: Supplementary file 1 [file Image_1.jpg]
